# Unlocking the Multifaceted Mechanisms of Bud Outgrowth: Advances in Understanding Shoot Branching

**DOI:** 10.3390/plants12203628

**Published:** 2023-10-20

**Authors:** Yundong Yuan, Said Khourchi, Shujia Li, Yanfang Du, Pierre Delaplace

**Affiliations:** 1 National Key Laboratory of Wheat Improvement, College of Life Sciences, Shandong Agricultural University, Tai’an 271018, China; 2 Plant Sciences, TERRA Teaching and Research Center, Gembloux Agro-Bio Tech, University of Liège, 5030 Gembloux, Belgium; 3 Institute of Genetics and Developmental Biology, Chinese Academy of Sciences, Beijing 100101, China

**Keywords:** bud outgrowth, tillering, branching, plant hormones, axillary meristem (AM), sugars, light, temperature, water, nutrients, biotic stresses

## Abstract

Shoot branching is a complex and tightly regulated developmental process that is essential for determining plant architecture and crop yields. The outgrowth of tiller buds is a crucial step in shoot branching, and it is influenced by a variety of internal and external cues. This review provides an extensive overview of the genetic, plant hormonal, and environmental factors that regulate shoot branching in several plant species, including rice, Arabidopsis, tomato, and wheat. We especially highlight the central role of *TEOSINTE BRANCHED 1* (*TB1*), a key gene in orchestrating bud outgrowth. In addition, we discuss how the phytohormones cytokinins, strigolactones, and auxin interact to regulate tillering/branching. We also shed light on the involvement of sugar, an integral component of plant development, which can impact bud outgrowth in both trophic and signaling ways. Finally, we emphasize the substantial influence of environmental factors, such as light, temperature, water availability, biotic stresses, and nutrients, on shoot branching. In summary, this review offers a comprehensive evaluation of the multifaced regulatory mechanisms that underpin shoot branching and highlights the adaptable nature of plants to survive and persist in fluctuating environmental conditions.

## 1. Introduction

The plasticity exhibited by plants in their shoot development is remarkable, as it allows them to adapt to various harmful external and internal conditions in order to survive and thrive. Shoot architecture in seed plants is primarily determined by factors such as the number, position, orientation, and size of shoot branches. The regulation of shoot branching/tillering constitutes a critical survival and propagation strategy governed by a complex, sophisticated regulatory network.

Initiation of the primary shoot axis can be traced back to the shoot apical meristem (SAM), a group of mitotic cells that forms during embryogenesis. Subsequently, the derivatives of this meristem give rise to all above-ground parts of plants [1]. The SAM produces aerial organs by continuously adding growth units called phytomers, generally comprising three parts: an internode, a leaf, and an axillary meristem (AM) that emerges at the leaf axil [2].

**Figure 1:**
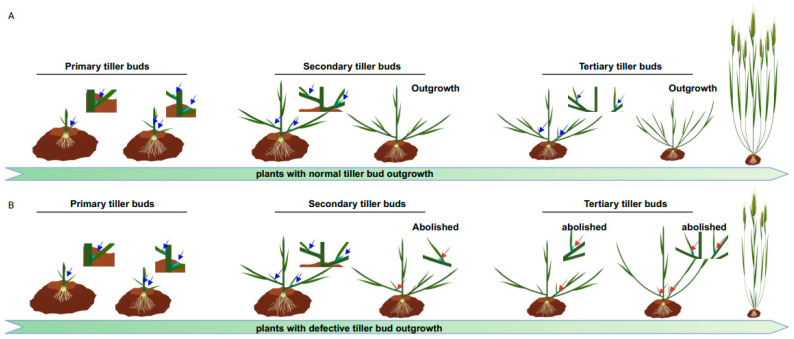
Illustration of the dynamic process of tiller formation and comparisons between plants with normal and defective tiller bud outgrowth. (**A**) portrays the successive processes of tiller bud formation and their outgrowth to generate more panicles than in (**B**). The primary tiller buds (arrows indicated) arise from the leaf axils of the main stem. Secondary tiller buds occur from leaf axils of the primary tillers and so on. We have highlighted with red arrows the abolished tiller buds that cannot grow to form tillers in (**B**).

AMs are new stem cell niches derived from the SAM during post-embryonic development. AM activity plays a vital role in generating the intricate branching patterns that contribute to a plant’s fractal architecture. Given its significant influence on shoot branching/tillering and panicle branching, the AM has been a focal point in breeding selection for improving crop production and management [3,4,5]. For the convenience of readers to understand the influence of crop yield, please refer to Figure 1 and Supplementary Video S1, which dynamically indicates the process of AMs’ outgrowth and their impact on grain yield.

**Figure 2:**
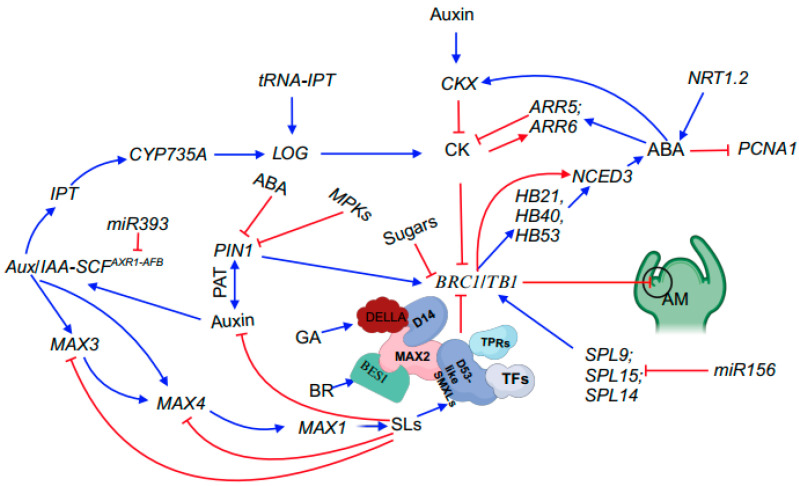
Summary of phytohormones and key genes involved in shoot branching. Blue arrows represent promotion, whilst red flat-ended lines denote inhibition. In this model, *BRC1/TB1* acts as an integrator to interact with other genes, such as *SPL* genes and phytohormones, to mediate shoot branching. Abbreviations: SL, strigolactone; ABA, abscisic acid; CK, cytokinin; GA, gibberellin; *PIN1*, *PIN-FORMED1*; *SCF*, *Skp-Cullin-F-box*; *Aux/IAA*, *Auxin*/*Indole-3-Acetic Acid*; *IPT*, *ADENYLATE ISOPENTENYLTRANSFERASE*; PAT, polar auxin transport; *CYP735A*, *cytochrome P450 monooxygenase 735A*; *LOG*, *LONELY GUY*; *tRNA-IPT*, *transfer RNA isopentenyltransferase*; *CKX*, *cytokinin oxidase*; *ARR5* and *ARR6*, *RESPONSE REGULATOR5* and *6*; *NRT1.2*, *nitrate transporter 1.2*; *PCNA1*, *PROLIFERATING CELL NUCLEAR ANTIGEN1*; *SPL9*, *SPL14* and *SPL15*, *SQUAMOSA PROMOTER BINDIN PROTEIN-LIKE9*,*14* and *15*; *BRC1/TB1*, *BRANCHED1/TEOSINTE BRANCH 1*; *NCED3*, *9-CIS-EPOXYCAROTENOID DIOXYGENASE3*; *HB21*, *HOMEOBOX PROTEIN 21*; *HB40*, *HOMEOBOX PROTEIN 40*; *HB53*, *HOMEOBOX PROTEIN 53*; DELLA, aspartic acid–glutamic acid–leucine–leucine–alanine; D14, DWARF 14; *MAX3*, *2*, *1*, and *4*, *more axillary growth 3*, *2*, *1*, and *4*; TPRs, TOPLESS-RELATED PROTEINs; TFs, transcription factors; D53-like SMXLs, DWARF53-LIKE SMAX1-LIKEs; BES1, bri1-EMS-suppressor 1; AM, axillary meristem.

Numerous studies conducted over the past decades have aimed to unravel the mechanisms underlying shoot branching. The prevailing understanding is that various inputs, such as endogenous factors, developmental cues, and environmental signals, interlock to regulate shoot branching. For instance, among multiple essential genes controlling shoot branching in plants, *TEOSINTE BRANCHED 1* (*TB1*) acts locally in buds to inhibit bud outgrowth and is considered to be an integrator of diverse phytohormonal, trophic, and environmental signaling networks (Figure 2) [6,7]. In addition, the signals defined by phytohormones, nitrogen, light, and sugars have been shown to significantly affect shoot branching (Figure 2) [8,9,10,11,12,13,14,15,16,17,18,19,20,21].

Significant advances have been made in our understanding of shoot branching, and numerous essential genes described in the literature have been demonstrated to influence shoot branching. However, the underlying mechanisms involving these genes are complicated, such as the effect of plant resistance genes on branching/tillering (in addition to resistance) when disrupted [22,23]. This review aims to provide a comprehensive summary of recent advances in our understanding of shoot branching, with a primary focus on Arabidopsis (*Arabidopsis thaliana*), wheat (*Triticum aestivum*), tomato (*Solanum lycopersicum*)*,* and rice (*Oryza sativa*). Elucidating and differentiating the complex mechanisms underlying shoot branching will contribute to the field of crop breeding, as shoot branching/tillering crucially determines plant architecture, which directly influences yield and overall productivity.

## 2. Mechanisms Regulating Shoot Branching

Once a branch/tiller bud is formed, the plant is confronted with a critical decision to stimulate bud sprouting, giving rise to a branch/tiller, or to maintain bud dormancy. Bud outgrowth typically progresses through three discernible stages: dormancy, transition, and sustained growth [24,25,26]. The fate of buds in the transition stage is influenced by the complex interplay of environmental and endogenous cues, ultimately determining whether buds return to dormancy or enter a sustained growth phase [27]. Consequently, the final count of branches/tillers is not solely determined by the number of axillary buds but is also influenced by the potential of buds to undergo growth [28]. In the following sections, we primarily focus on elucidating shoot branching through the lens of endogenous cues and environmental signals. For a visual representation of the interplay among various components, please refer to the conceptual model of bud outgrowth shown in Figure 2. The genes mentioned in this section are referred to in Table 1.

### 2.1. Internal Inputs Determine Bud Outgrowth

#### 2.1.1. TB1/BRC1 Acts as a Key Integrator of Branching

The expression of *TB1*, encoding a non-canonical basic helix–loop–helix (bHLH) transcription factor of the TCP family, is negatively correlated with bud growth [63,64]. This TCP protein family is represented by four founding members: TB1, CYCLOIDEA (CYC), PROLIFERATING CELL NUCLEAR ANTIGEN FACTOR1 (PCF1), and PCF2. These family members were identified by their functions in plant development or their DNA binding capacity [63,65,66,67,68]. The maize (*Zea mays*) *tb1* mutant exhibits an uncontrolled proliferation of tillers, resulting in a bushy architecture reminiscent of its ancestor, teosinte [29]. The inhibitory effect of *TB1* on bud outgrowth is spatially restricted to axillary buds as soon as they become visible [29]. By contrast, in teosinte, *TB1* is not expressed in axillary buds, allowing axillary bud outgrowth [29]. The role of *TB1* in suppressing axillary bud outgrowth is conserved in rice, as ectopic overexpression of its ortholog *OsTB1* under the control of the actin promoter leads to reduced tillering [69]. Conversely, loss-of-function mutants of *OsTB1*, such as *fine culm 1* (*fc1*), show increased tillering [69]. *TB1* and its orthologs (e.g., *OsTB1* or *FC1* in rice, *BRANCHED1* (*BRC1*) in Arabidopsis, *PsBRC1* in pea [*Pisum sativum*], and *SlBRC* in tomato) operate in conjunction with other vital genes and plant hormones to regulate bud outgrowth [6,30,31,70]. These genes and phytohormones are discussed in subsequent sections. The coordinated action of TB1 with these factors has earned it the title “branching integrator” (Figure 2).

It is worth noting that several studies have indicated that inhibition of bud outgrowth can occur independently of *TB1* and its orthologs [2,31,71,72]. Hence, *TB1* may condition bud activation potential, thus contributing to the regulation of branching.

#### 2.1.2. SQUAMOSA Binding Proteins Inhibit Bud Outgrowth

SQUAMOSA promoter binding protein-like (SPL) transcription factors, which are specific to plants, mediate various aspects of plant development, including branching [32]. Different members of the *SPL* gene family in Arabidopsis are post-transcriptionally regulated by miR156 [73]. Overaccumulation of miR156 leads to a considerably bushy phenotype [74,75]. Notably, double mutants of the Arabidopsis paralogs *SPL9* and *SPL15* exhibit an increased branching phenotype, highlighting the crucial role of miR156-targeted *SPL* genes in regulating shoot branching [33] (Figure 2). Accumulation of the SPL9 and SPL15 ortholog OsSPL14 results in fewer tillers and increased yield in rice [5]. These findings highlight the roles of SPL proteins in inhibiting branching. Additionally, OsSPL14, whose encoding transcripts are targeted by miR156, directly activates *OsTB1* expression [5].

#### 2.1.3. Auxin Indirectly Inhibits Sustained Bud Outgrowth

A principal function of auxin, as observed in various studies, is mediating apical dominance, as lateral bud outgrowth is inhibited by auxin. For instance, the *auxin-resistant 1* (*axr1*) mutant of Arabidopsis exhibits a lower sensitivity to auxin than wild-type plants, resulting in weak apical dominance and an increased number of branches, due to the release of axillary buds, highlighting the role of auxin in inhibiting axillary bud outgrowth [34]. Conversely, auxin-overproducing mutants with elevated levels of free auxin, such as the Arabidopsis *yucca* mutants, exhibit stronger apical dominance than wild-type plants [35].

Moreover, apical dominance largely depends on polar auxin transport (PAT) mediated by PIN-FORMED (PIN) proteins in the stem [76]. Studies involving RNA interference (RNAi)-mediated knockdown and overexpression of *OsPIN1* have demonstrated the negative effect of *OsPIN1* on tillering in rice [36]. In addition, overexpression and knockdown experiments with *OsPIN5B* revealed the influence of this gene on tiller numbers [37]. Mitogen-activated protein kinase (MAPK) cascades are essential for transducing external and internal cues into adaptive and programmed responses. The MKK7 (MAPK KINASE 7)-MPK6 signaling pathway regulates PAT by phosphorylating the specific substrate PIN1, thereby modifying shoot branching in Arabidopsis [38,39].

In addition to the crucial role of PAT in regulating branching/tillering, the auxin signaling pathway also influences shoot branching/tillering via SKP1-CULLIN1-F-box (SCF)-mediated protein degradation. Auxin receptors, including TRANSPORT INHIBITOR RESPONSE 1 (TIR1) and closely related family numbers (AUXIN SIGNALING F-BOX [AFB]) [9,40,41], bind to auxin to stabilize the interactions between TIR1/AFBs and members of the Aux/IAA (Auxin/INDOLE-3-ACETIC ACID INDUCIBLE) family of transcriptional repressors [77]. Their interaction with TIR1/AFBs leads to the degradation of Aux/IAA, permitting auxin-mediated upregulation of transcription [78,79,80]. By contrast, loss of function of *IAA12* in Arabidopsis leads to auxin-resistant stabilization of the SCF complex and, thus, constitutive suppression of target auxin-upregulated genes [41], resulting in a bushy phenotype. Overexpressing *OsMIR393*, whose mature miRNA product OsmiR393 targets and downregulates the transcripts of the auxin receptor genes *OsTIR1* and *OsAFB2*, leads to increased tiller production [42].

Moreover, many *Aux/IAA* genes are rapidly transcriptionally induced by auxin in a SCF^TIR/AFB^-dependent manner [81]. *TIR1/AFB* genes encode F-box proteins that interact with the cullin CUL1 and the SKP1-like proteins ASK1 or ASK2 to form an SCF ubiquitin protein ligase (E3). TIR1/AFB genes function as transcription factors that bind to auxin-response elements (AuxREs) located in the upstream regions of auxin-inducible genes [82]. Auxin resistant 1 (AXR1) is required for proper SCF function as it facilitates conjugation of the ubiquitin-like protein RELATED TO UBIQUITIN 1 (RUB1) to the cullin subunit [9,83,84]. Correspondingly, mutations in *AXR1* result in changes in the expression of SCF^TIR1/AFB^-dependent auxin-responsive genes, leading to defects in downstream auxin responses [9] (Figure 2).

It should be noted that the effect of auxin on branching/tillering is indirect, as apically derived auxin cannot enter buds [85]. Two primary models have been proposed to explain this phenomenon: the canalization model and the second-messenger-based model. According to the canalization model, axillary buds are activated when the amount of auxin initially flowing out of the bud is sufficient to trigger the establishment of polar auxin transport connected to the auxin stream in the stem, thereby promoting bud outgrowth [86,87]. Conversely, the continuous flow of auxin in the stem originating from the apex restricts the export of auxin from the axillary buds on the same axis, thereby maintaining apical dominance [88]. The establishment of auxin transport involves a positive regulatory feedback loop between the polarization of auxin efflux-facilitating PINs at the plasma membrane in the direction of the initial flow and the directional flow [89]. In addition, strigolactones (SLs) act upstream of auxin by stimulating the removal of PIN1 from the plasma membrane, thereby reducing the ability of the bud to create its own polar auxin transport [90]. According to the second-messenger-based model, auxin flow in the main stem negatively modulates cytokinin (Ck) biosynthesis [52] and positively regulates SL levels [91], with these two phytohormones acting antagonistically on buds by inducing and inhibiting their outgrowth, respectively [92,93]. Furthermore, the antagonistic effects of Cks and SLs in buds are integrated by *BRC1*, the Arabidopsis homolog of *TB1* that is mainly expressed in dormant axillary buds [6,31,93].

The transition of a bud from dormancy or quasi-dormancy to more active outgrowth is associated with increased expression of genes involved in the cell cycle [25,94,95]. Expression of cell cycle-related genes is repressed by auxin biosynthesized in the shoot apex, leading to suspension of cell division and bud dormancy [25].

#### 2.1.4. Strigolactones Have an Inhibitory Effect on Bud Outgrowth

SLs are a collection of carotenoid-derived lactones secreted by plants. These phytohormones are primarily known for their roles as rhizosphere signals used by root-parasitic plants to detect their hosts [96] and as cues for mycorrhizal fungi to form symbiotic associations [97]. Importantly, SLs also inhibit the outgrowth of axillary buds. This inhibitory effect was initially observed in *ramosus* (*rms*) mutants in pea, which exhibit excessive branching, as well as *decreased apical dominance* (*dad*) mutants in petunia (*Petunia hybrida*) and *more axillary growth* (*max*) mutants in Arabidopsis [45,98,99,100,101,102,103,104].

SL biosynthesis involves several enzymes. DWARF27 (D27) is responsible for isomerizing all-*trans*-β-carotene at the C-9 position to form 9-*cis*-carotene [12,43]. Carotenoid cleavage dioxygenase 7 (CCD7, also named MAX3) and CCD8 (also named MAX4) then cleave 9-*cis*-carotene to produce carlactone, a key endogenous SL precursor [44,45]. The conversion of carlactone to SLs is catalyzed by MAX1, an Arabidopsis cytochrome P450 that acts downstream of MAX4 and MAX3 [46]. MAX1 converts carlactone to carlactonoic acid, which is further converted to methyl carlactonoate, an SL-like compound (Figure 2) [105].

SLs are perceived by MAX2, an ortholog of D3 from rice, which plays a crucial role in regulating plant branching. Disruption of *MAX2* leads to a bushy phenotype in Arabidopsis [46]. Moreover, the perception and signaling roles of SLs in rice require their interaction with D14, a putative SL receptor. D14 interacts with D3, an F-box protein of the SCF E3 ubiquitin ligase complex, to form an SL-induced D14-D3 complex [47]. This complex targets proteins for ubiquitination and degradation, resulting in changes in plant branching/tillering [48] (Figure 2).

The dominant *d53* mutant in rice is characterized by a high-tillering and dwarf phenotype and is resistant to the exogenous application of GR24, a synthetic SL. D53 is targeted for SL-dependent degradation by the SCF^D3^ ubiquitination complex [106]. In addition, SL treatment results in D53 degradation via the ubiquitin–proteasome system in a D14- and D3-dependent manner [48]. D53 interacts with members of the TOPLESS-RELATED PROTEIN (TPR) family of transcriptional co-repressors, which may suppress the activities of their downstream transcription factors (Figure 2) [47,107].

In Arabidopsis, D53-like proteins, including SMAX1-LIKE6 (SMXL6), SMXL7, and SMXL8, are targeted for proteolysis by MAX2-mediated SL signaling [48,49]. Their overaccumulation in SL signaling mutants (such as *max2*) leads to increased branching and constitutively low *BRC1* expression in buds. Conversely, disruption of *SMXL6*, *SMXL7*, and *SMXL8* completely restores branching of *max2* to wild-type levels and leads to very high levels of *BRC1* transcript in inhibited buds [48,49]. This effect highlights the role of SLs in inhibiting bud outgrowth by upregulating *BRC1* transcription. Additionally, these SMXL proteins can form a complex with TPR2 and function as transcriptional repressors. However, D14 interacts with SMXLs and MAX2 in an SL-dependent manner, thereby inhibiting axillary bud outgrowth in Arabidopsis [48].

The relationship between SLs and *BRC1* and its corresponding orthologs suggests that the SL pathway acts upstream of *FC1/OsTB1* in rice. For example, the *fc1* mutant does not respond to application of GR24, and the phenotype of the *fc1 d17* double mutant is similar to that of the SL-deficient mutant *d17*, suggesting the involvement of SLs in regulating *FC1*/*OsTB1* expression in rice [70]. In line with this notion, *PsBRC1* expression levels are significantly lower in SL-deficient mutants (*rms1*, *rms2*, and *rms4*) than in wild-type pea plants [31] but are upregulated in *rms1* and *rms2* after GR24 application, further supporting the idea that SLs act upstream of *BRC1.* Specifically, *BRC1* is repressed in non-elongated *max2* and *max3* axillary buds but induced in the *smxl6 smxl7 smxl8*, *max2 smxl6 smxl7 smxl8*, and *max3-9 smxl6 smxl7 smxl8* mutants of Arabidopsis, suggesting that SMXL6, SMXL7, and SMXL8 inhibit *BRC1* expression [48].

#### 2.1.5. Other Phytohormones Regulate Tillering/Branching

The inhibitory role of auxin in bud outgrowth is exerted indirectly within buds, as apically derived auxin is not transported into buds [108] and exogenous auxin directly supplied to buds fails to prevent their growth [109]. Cytokinins (CKs) are believed to be crucial in relaying the auxin signal into buds and promoting axillary branching. CKs are an important class of phytohormones that participate in various aspects of plant development, including organ formation, apical dominance, and leaf senescence [110]. Therefore, CKs facilitate the growth and development of axillary buds by serving as a second messenger for the auxin signal.

Several studies have demonstrated that CKs can promote the outgrowth of buds that would otherwise remain inhibited and that CK levels in or near the bud are well correlated with bud fate [50,111]. For example, in chickpea (*Cicer arietinum* L.) plants, CK levels dramatically increased in axillary buds within 24 h of shoot decapitation [112]. Elevated levels of CKs, as achieved by overexpressing *ISOPENTENYL TRANSFERASE* (*IPT*) (encoding a key enzyme in CK biosynthesis), lead to reduced apical dominance (Figure 2) [50]. Continuous treatment of pea plants with synthetic CKs overcomes the inhibition of lateral bud release, turning these into dominant organs [113]. In Arabidopsis, basally applied CKs suppress the inhibitory effects of apically supplied auxin in isolated nodes [114]. Conversely, low local CK levels can limit bud outgrowth, even in auxin- and strigolactone-deficient plants [115]. The *supershoot* (*sps*) mutant of Arabidopsis displays overproliferating branching due to cytokinin-promoted bud initiation as well as bud outgrowth, as endogenous cytokinin levels are elevated in this mutant [51]. The Arabidopsis *altered meristem program 1* (*amp1*) mutant, with increased cytokinin levels, shows enhanced AM formation and bud elongation to generate branches [8]. Interestingly, in this mutant, *BRC1* is slightly downregulated, suggesting that Cks downregulate *BRC1* expression [6].

In addition to their biosynthesis, the metabolism of CKs also determines endogenous cytokinin levels. Cytokinin oxidase (CKX) is the enzyme responsible for inactivating CKs by irreversibly degrading active CKs, thereby regulating endogenous levels of active CKs [116,117]. Various CKXs, such as PsCKX2 in pea, predominantly regulate CK levels in the stem [52]. Notably, the expression pattern of *PsCKX2* in the stem is opposite to that of CK levels before and after decapitation, suggesting that *PsCKX2* induces a decrease in CK levels in the stem. Overall, these findings support the notion that CKs promote bud outgrowth.

While abscisic acid (ABA) is predominantly recognized for its roles in seed dormancy, growth inhibition, and stress responses, emerging evidence indicates that this phytohormone also influences plant branching/tillering. Mutations in key genes involved in ABA biosynthesis, such as *9-CIS-EPOXYCAROTENOID DIOXYGENASE3* (*NCED3*) and *ABA DEFICIENT2* (*ABA2*), enhance bud outgrowth [53,54]. Notably, ABA levels are elevated in buds with delayed outgrowth but are reduced in elongated buds [54]. Exogenous application of ABA partially suppresses branch elongation, suggesting that ABA functions downstream or independently of genes responsible for bud growth [54]. Unlike the IAA biosynthesis gene *TRYPTOPHAN AMINOTRANSFERASE OF ARABIDOPSIS 1* (*TAA1*) and the auxin transporter gene *PIN1*, exogenously supplied ABA does not affect *BRC1* expression, confirming the downstream role of ABA in regulating *BRC1*. Furthermore, BRC1 binds to the promoter of and positively regulates the transcription of three genes encoding related homeodomain leucine zipper proteins (HD-ZIP): *HOMEOBOX PROTEIN 21* (*HB21*), *HB40*, and *HB53*. Together with BRC1, these three proteins promote *NCED3* expression, resulting in ABA accumulation and triggering a phytohormonal response, thereby suppressing bud development [14] (Figure 2). Finally, ABA treatment represses the expression of the cell cycle-related gene *PROLIFERATING CELL NUCLEAR ANTIGEN1* (*PCNA1*), suggesting that ABA modulates bud outgrowth by regulating cell cycle-related gene expression [54].

Gibberellins (GAs) are renowned for their ability to regulate internode elongation. Dwarfism is often associated with an increase in shoot branching. GA-deficient mutants in Arabidopsis, rice, and pea exhibit higher levels of branching/tillering than their wild-type counterparts [10,118]. Likewise, overexpressing GA catabolism genes decreases GA levels, resulting in increased branching/tillering [10]. Moreover, mutants with disruption of DELLA proteins, the major negative regulators of GA signaling, display reduced shoot branching and/or altered branching patterns [32]. Additionally, in rice, DELLA SLENDER RICE 1 (SLR1) was found to interact with the SL receptor D14 in an SL-dependent manner [32] (Figure 2).

Brassinosteroids (BRs) are important plant hormones that regulate various developmental processes, including stem elongation, leaf development, senescence, and branching [119]. A series of BR-deficient mutants have been used to elucidate the function of BR. For instance, reduced expression of *Brassinazole Resistant 1* (*OsBZR1*) in rice leads to a dwarf phenotype with erected leaves and reduced BR sensitivity [60]. *Dwarf and Low Tillering* (*DLT*), encoding a GRAS family protein, is another BR-related gene. Disruption of *DLT* results in a semi-dwarf mutant with fewer tillers and decreased BR responses. The promoter of *DLT* can be targeted by *OsBZR1* [61]. *GLYCOGEN SYNTHASE KINASE 2* (*GSK2*) encodes a conserved glycogen synthase kinase 3-like kinase. Gain-of-function mutations within the *GSK2* coding sequencing or its overexpression suppress BR signaling, leading to plants with phenotypes resembling BR-deficient mutants [120]. The *reduced leaf angle 1* (*rla1*) mutant encodes a transcription factor containing an APETALA2 (AP2) DNA binding domain that is required for *OsBZR1* function [62]. RLA also can interact with GSK2 [62].

A BR-defective rice mutant displays reduced branching like *dlt* [61], while in Arabidopsis, the *bri1-EMS-suppressor 1* (*bes1*) mutant displays a highly branched phenotype. In contrast, *BES1*-RNAi lines have fewer branches than the wild type [55,119]. Interestingly, the dominant *bes1-D* mutant does not respond to GR24 treatment, and BES1 can interact with MAX2 and act as its substrate for degradation, which is regulated by SLs [15]. Therefore, SL and BR signaling pathways converge on the same transcription factor BES1, an Arabidopsis homolog of OsBZR1, to control branching [15]. However, other components upstream of *AtBES1* in the BR signaling pathway do not alter branching in Arabidopsis [15]. In rice, BRs bind to the receptor *Brassinosteroid insensitive 1* (*BRI1*), activating the receptor complex and inhibiting the ability of OsGSK2 [120] to phosphorylate members of the downstream transcriptional module, including OsBZR1, DLT, and RLA1, and thereby regulate their stability [62]. Furthermore, BRs strongly enhance tillering by promoting bud outgrowth in rice through regulating the stability of D53 and/or the *OsBRZ1*-*RLA1*-*DLT* module, a transcriptional complex in the BR signaling pathway [15]. In addition, D53 interacts with OsBZR1 to inhibit the expression of *OsTB1*. This interaction depends on direct DNA binding by OsBZR1, which recruits D53 to the *OsTB1* promoter in axillary buds [15].

#### 2.1.6. Phytohormones Interact Influencing Bud Outgrowth

The regulation of axillary bud outgrowth involves a complex network of phytohormones. Plant hormones, including auxin, CKs, and SLs, play central roles in bud outgrowth. Furthermore, plant hormones can mutually affect each other, ultimately regulating branching/tillering.

Auxin is an indispensable player in plant architecture, particularly branching, and a central component of interacting networks that regulate branching. Auxin exerts its role indirectly in buds [85], whereas SLs are direct components that act on buds via auxin to inhibit bud outgrowth. Auxin inhibits bud outgrowth by regulating SL biosynthesis, as observed in Arabidopsis and rice [92,121,122]. For example, auxin that originates in the shoot apex modulates SL levels in pea by maintaining *RMS1* and *RMS5* transcript abundance [123,124]. The *iaa12* mutant exhibits increased numbers of branches and shows reduced expression of the SL biosynthesis genes *MAX3* and *MAX4* [92], while *MAX3* and *MAX4* transcription is mediated by auxin [125]. Conversely, Arabidopsis mutants defective in SL biosynthesis (such as *max4*) exhibit increased branching and resistance to auxin [45]. Additionally, branching is inhibited by SL treatment in auxin-response mutants such as *axr1* and the *tir1 afb1 afb2 afb3* quadruple mutant [126], providing compelling evidence that loss of auxin signaling promotes bud outgrowth via SL depletion. Moreover, both auxin biosynthetic and auxin signaling mutants respond to SL treatment, indicating that SLs function downstream of auxin [91].

SLs also inversely affect polar auxin transport by limiting the accumulation of the auxin efflux carrier PIN1 in cells involved in polar auxin transport [87]. The Arabidopsis *max* mutants, with defects in the SL pathway, show enhanced polar PIN accumulation and auxin transport [127,128,129]. Similarly, the rice *d27* mutant (characterized by low SL concentrations) displays enhanced auxin transport [12]. Therefore, auxin and SLs interact via interconnected feedback loops, where each phytohormone regulates the level of the other.

Auxin and CKs play antagonistic roles in regulating bud outgrowth. Auxin inhibits AM outgrowth, whereas CKs counteract auxin activity in Arabidopsis by promoting bud activation [20]. The inhibitory effect of auxin is probably mediated, at least in part, by its ability to reduce both CK export from roots and CK biosynthesis locally at the node [130,131]. Stem girdling in pea, which prevents polar auxin transport via a mechanism similar to decapitation, increases the expression of CK biosynthesis genes and promotes the growth of buds below the girdling site [115]. Decapitated bean (*Phaseolus vulgaris* L.) plants (with a decrease or loss of polar auxin transport) have higher CK concentrations in xylem exudates than control plants. However, applying auxin to the shoots of decapitated plants eliminates the effect of shoot-tip removal on CK concentration, further supporting the antagonistic relationship between auxin and CKs [130]. Auxin inhibits expression of the CK biosynthesis gene *IPT* in the stem [111,115,130,132]. In Arabidopsis, auxin-mediated suppression of CK biosynthesis is dependent on AXR1 [131]. However, in addition to repression by auxin, CKs also enhance auxin production and promote downward auxin transport out of growing buds. This, in turn, suppresses the production of CKs lower within the stem, limiting their accessibility to other buds [52,111,133].

In addition to the inhibitory roles of auxin on CKs, ABA represses CK signaling by inducing the expression of type-A response regulator genes, such as *RESPONSE REGULATOR5* (*ARR5*) and *ARR6*, encoding negative regulators of CK signaling [134] (Figure 2). Moreover, ABA inhibits the accumulation of CKs in the roots and shoots of wheat and promotes CKX activity, contributing to the decrease in CK levels [135]. Conversely, overexpression of *IPT* results in reduced ABA abundance in petunia flowers [136]. ABA suppresses auxin biosynthesis and auxin transport out of axillary buds [54].

#### 2.1.7. Sugars Play an Essential Role in Bud Release

Sugars such as sucrose serve not only as a carbon source for plant metabolism but also as essential signaling compounds [137,138,139]. Bud outgrowth responds to limiting nutrients and resources, and thus sugars have been attracting increasing interest. Axillary buds are often maintained in a dormant state or their growth is suppressed by the growing shoot apex long after their initial formation. Intriguingly, changes in sugar availability can facilitate the first visible growth of buds, a phenomenon known as axillary bud release [16]. The term “apical dominance” is commonly used to describe shoot branching, referring to the role of the shoot tip in preventing the growth of the axillary buds below it [140]. However, in several plant species, auxin supplementation to the decapitated stump, even at high levels, fails to fully restore apical dominance [109,141]. Therefore, it is unlikely that auxin is the first component inhibiting axillary bud outgrowth. Rather, after decapitation, sugars are rapidly redistributed over a significant distance and accumulate in axillary buds, coinciding with the timing of bud release, before auxin depletion occurs in the corresponding axillary buds [16,142]. This observation suggests that sugars have the potential to promote bud release.

Enhancing the sugar supply alone is sufficient for bud release. Plants employ various mechanisms to maintain apical dominance, one of which is limiting the sugar supply to axillary buds [16]. This effect can be observed in the wheat *tiller inhibition* (*tin*) mutant, whose reduced tillering is associated with a decreased sucrose content in axillary buds [143]. Likewise, reduced tiller formation in the rice *monoculm 2* (*moc2*) mutant is attributed to a disruption in fructose-1,6-bisphosphatase, an enzyme involved in sucrose biosynthesis, resulting in a decline in the sucrose supply [56]. The requirement for sugars for bud outgrowth has been demonstrated in rose (*Rosa hybrida*), where sugar is required for triggering bud outgrowth in single nodes cultivated in vitro [88,144]. Sucrose can also modulate the dynamics of bud outgrowth in a concentration-dependent manner, especially during the transition phase between bud release and sustained bud elongation [88]. Additionally, removal of competing sugar sources or sinks within buds through defoliation further supports the role of sugars in bud release [16,145]. Collectively, these lines of evidence highlight the trophic role of sugars in bud release.

Besides the roles of sugars as nutrients, an effect of sugars on phytohormone homeostasis has been demonstrated in single nodes of *R. hybrida* [88]. For instance, sucrose stimulates CK biosynthesis in bud-bearing stem segments by upregulating the expression of two CK biosynthesis-related genes [88]. Sucrose can also modulate auxin metabolism, as treatment with sucrose or its non-metabolizable analogs increases auxin levels in *R. hybrida* buds in a concentration-dependent manner [88]. Furthermore, elevated sucrose levels in buds promote the export of auxin from the bud to the stem, which is favorable for bud outgrowth according to the auxin canalization model [88]. When exogenously supplied to rose, sucrose reduces the expression of *MAX2*, a gene involved in SL signaling [88]. A dose-dependent inhibitory effect by sucrose has also been detected for *RhBRC1* expression, which is decisive in preventing bud outgrowth [31,146]. Notably, palatinose, a non-metabolizable sucrose analog, can trigger bud outgrowth [144]. These findings collectively demonstrate the crucial role of sugar signaling in regulating bud release.

From a trophic perspective, axillary buds are sink organs that require imported sugars to fulfill their metabolic demands and support their growth. In the context of apical dominance, the supply of sugars to lateral buds is the first signal that releases bud dormancy, preceding detectable auxin depletion. Thus, the growth potential of a bud can be determined by its sink strength, representing its ability to acquire and utilize sugars. The interplay in the demand for sugar between the apical bud and lateral buds is thus crucial for the systemic regulation of shoot branching, encompassing both nutritional support and signaling mechanisms.

### 2.2. Effects of Environmental Inputs on Bud Outgrowth

Bud outgrowth and the transition to dormancy are tightly regulated by various environmental factors, including photoperiod, light intensity, nutrient availability, and stress conditions [26,147]. Notably, ABA, a pivotal phytohormone involved in regulating bud outgrowth, strongly accumulates under stressful conditions such as osmotic stress [148]. Interestingly, ABA levels are elevated in buds with delayed outgrowth but are reduced in elongated buds [54]. This dynamic modulation of ABA levels underscores the remarkable ability of plants to adjust their branching capability in response to diverse environmental cues. Such adaptability represents a successful evolutionary trait that has evolved to accommodate the sessile nature of plants. Here, we focus on environmental cues reported so far. The genes mentioned in this section are referred to in Table 2.

#### 2.2.1. Light Plays a Critical Role in Bud Outgrowth

Light intensity is pivotal for regulating bud outgrowth across numerous plant species. For instance, low-intensity light inhibits tillering in wheat [163]. By contrast, high-intensity light stimulates branching, as observed in *Rosa* species [164].

Photoperiod also has a significant influence on the distribution of bud outgrowth along the plant stem. For example, under short-day conditions, the formation of basal branches is enhanced in pea. Bud outgrowth in the upper nodes often coincides with the onset of flowering and may also be controlled by photoperiod [165,166].

**Figure 3:**
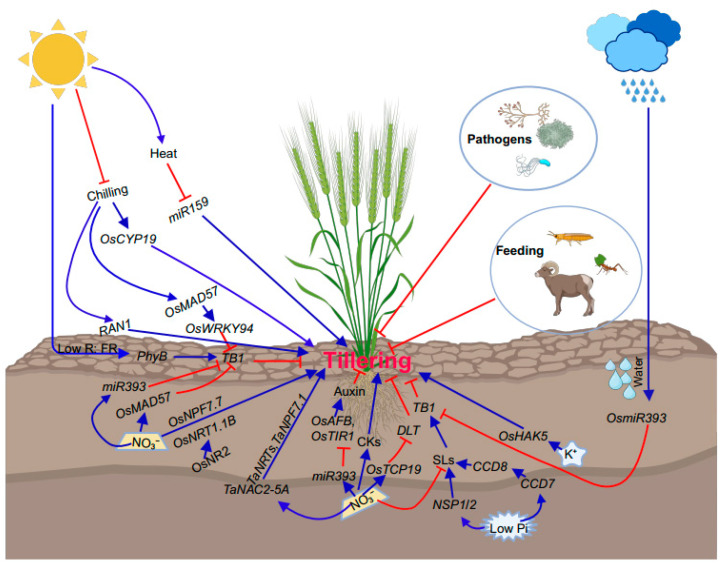
Effects of environmental inputs on bud outgrowth. The roles of various environmental factors are indicated, and genes involved are depicted. Blue arrows represent promotion, while red-flat-ended lines denote inhibition. In this model, the abiotic factors like nutrients, light, temperature, and biotic stresses impact tillering significantly. Abbreviations: *OsCYP19*, *Oryza sativa cytochrome P450 family 19; OsMAD57*, *Oryza sativa MADS-box protein 57*; *miR159*, *microRNA 159; PhyB*, *phytochrome B*; *TB1*, *TEOSINTE BRANCHED 1*; *OsNPF7.7*, *peptide transporters family 7.7*; *OsNRT1.1B*. Oryza sativa *Nitrate Transporter 1.1B*; *OsNR2*, *Oryza sativa NADH/NADPH-DEPENDENT NITRATE REDUCTASE 2*; *OsWRKY94*, *Oryza sativa WRKY transcription factor 94; TaNAC2-5A*, *Triticum aestivum NAC transcription factor 2-5A*; *miR393*, *microRNA 393*; CK, cytokinin; SL, strigolactone; *OsAFB*, *Oryza sativa AUXIN SIGNALING F-BOX 2*; *OsTIR1*, *Oryza sativa TRANSPORT INHIBITOR RESPONSE*; *OsTCP19*, *TEOSINTE BRANCHED1/CYCLOIDEA/PROLIFERATING CELL FACTOR 19*; *DLT*, *DWARF AND LOW-TILLERING*; *NSP1*/*2*, *Nodulation Signaling Pathway 1*/2; Pi, phosphorus; *OsHAK5*, *Oryza sativa High-Affinity* K^+^ *Transporter 5*; K^+^, potassium; *CCD7*/8, *Carotenoid cleavage dioxygenase 7*/*8*.

At high density, shade also regulates bud dormancy in cultivated plants [167]. When plants intercept incident light, the light intensity decreases, preferentially in the red part of the light spectrum. Shade is, therefore, characterized by a reduction in the red (R) to far-red (FR) light ratio (R:FR) due to R light absorption and FR reflection by leaves [168]. This decrease in R:FR serves as a signal of shade or competition for light, prompting plants to respond by inhibiting axillary bud outgrowth, elongating their stature, and accelerating flowering to evade the detrimental consequences of shading. This suite of responses, known as shade avoidance syndrome, is mediated by the R- and FR-absorbing photoreceptor phytochrome B (PHYB) [169]. In densely grown sorghum (*Sorghum bicolor*) plants experiencing shade, inhibition of bud outgrowth due to an enriched FR-light environment is associated with activation of the *TB1-like* gene *SbTB1* in buds [13,149]. Conversely, in the absence of shade, a higher proportion of phyB in the active form induces bud outgrowth by downregulating *SbTB1*. Shade signals with their low R:FR ratios decrease the proportion of the active form of phyB, thereby enhancing the expression of *SbTB1* and promoting bud dormancy (Figure 3). Interestingly, ABA also controls bud growth in response to R:FR shifts [53]. Furthermore, branching in response to a low R:FR ratio is defective in the ABA-deficient mutant *aba2-1* and the ABA biosynthetic mutant *nced3-2* [53].

#### 2.2.2. Impacts of Nutrients on Bud Outgrowth

Nitrogen (N) is an essential macronutrient that dominates plant growth and plant productivity [170,171]. N limitation can significantly limit the tiller numbers in rice [170]. Furthermore, high branching ability is positively correlated with the capacity for N uptake. Several N transporters have been identified that regulate shoot branching in rice. One such transporter is peptide transporters family 7.7 (OsNPF7.7), whose increased abundance facilitates the influx of NO_3_^−^ and NH_4_^+^, thereby promoting the outgrowth of axillary buds [57]. *Indica NADH/NADPH-DEPENDENT NITRATE REDUCTASE 2* (*OsNR2*) promotes NO_3_^−^ uptake through interaction with *OsNRT1.1B*, a low-affinity NO_3_^−^ transport gene, and increases effective tillers in *japonica* rice. Similarly, the *japonica* allele of *OsNR2* also promotes tillering but not to the extent observed in *indica OsNR2*-overexpression lines [150,172]. The rice APETALA2-domain transcription factor encoded by a *NITROGEN MEDIATED TILLER GROWTH RESPONSE 5* (*NGR5*) allele is upregulated under conditions of increased nitrogen availability. NGR5 interacts with a component of the polycomb repressive complex 2 (PRC2) to regulate the expression of *D14* and *OsSPL14* by mediating levels of histone methylation (H3K27me3) modification, thereby regulating rice tillering [151,172]. Overexpression of *DENSE AND ERECT PANICLE1* (*OsDEP1*) can increase tiller numbers under high N supply [152]. OsSPL14 can directly activate the expression of *OsDEP1* and *OsTB1* to regulate tiller bud outgrowth [173]. A microRNA, *miR393*, can target and repress the expression of *OsTB1* and the two auxin receptors *OsAFB2* and *OsTIR1* under NO_3_^−^ conditions, which influences the transport of auxin and eventually regulates tillering [153]. The rice *TEOSINTE BRANCHED1/CYCLOIDEA/PROLIFERATING CELL FACTOR 19* (*OsTCP19*) transcription factor can directly bind and repress the activity of the tiller-promoting *DLT* gene, thereby negatively regulating tillering in the presence of nitrogen (N). Further investigation revealed a 29-bp insertion/deletion polymorphism in the *OsTCP19* promoter that confers differential transcriptional response to N among rice varieties [154]. In wheat, the NO_3_^−^-inducible *CEREAL-SPECIFIC NAM*, *ATAF*, and *CUC* (*NAC*) transcription factor *TaNAC2-5A*, whose gene expression is induced by a limited NO_3_^−^ supply, directly binds to the promoters of genes encoding NO_3_^−^ transporters and glutamine synthetase, thereby enhancing N acquisition and assimilation. Notably, *TaNAC2-5A* overexpression leads to enhanced tiller numbers [58]. OsMADS57, a NO_3_^−^-inducible MADS-box transcription factor, interacts with OsTB1 and targets D14 to control the outgrowth of axillary buds in rice [59] (Figure 3).

Following decapitation, the primary forms of available and transported N are amino acids through the phloem, which might be easy for buds to obtain [17]. This notion is supported by the finding that the levels of three key amino acids, aspartate, asparagine, and glutamine, increase in axillary buds after decapitation, coinciding with the initiation of bud outgrowth. Interestingly, sucrose fails to trigger bud outgrowth in excised rose nodes in the absence of asparagine in the growth medium.

Given that N availability influences CK and SL levels, it is plausible that N functions as a second messenger to mediate branching/tillering [174,175,176]. In several species, limited N availability promotes SL production, subsequently inhibiting branching/tillering. N limitation also leads to a reduction in CK production. N fertilization can suppress the expression of SL biosynthesis genes [171,177]. Collectively, these findings highlight the important role of N (as with sugars) in influencing branching.

Phosphorus (Pi) is an essential macronutrient for plant growth and metabolism. However, the availability of Pi in soils is often low due to chemical fixation and poor diffusion, resulting in low-Pi environments that can limit plant development and processes like tillering [178]. For example, Pi deficiency has been shown to reduce tiller numbers in rice [179], with the transcription factor *Oryza sativa PHOSPHATE STARVATION RESPONSE2* (*OsPHR2*) implicated in the repression of tillering under low Pi conditions [155]. Pi deficiency also induced SL biosynthesis by increasing transcription of SL biosynthetic genes like the β-carotene *cis*-*trans isomerase DWARF27 (D27),* the *carotenoid cleavage dioxygenase 7 (CCD7)/D17*, and *CCD8/D10* [2,179,180], which play a key role in regulating tillering, as described above. Additionally, nodulation signaling pathway 1 (NSP1) and NSP2, two GRAS family transcription factors, have been shown to promote SL production in rice under low-Pi conditions by directly binding to SL biosynthetic gene promoters as a complex [156]. Moreover, Pi deficiency represses tiller numbers by promoting the degradation of D53 and the expression of *OsTB1*. Further examination of the mechanisms of genes in response to Pi availability will provide a deeper understanding of nutrient-mediated tillering.

Potassium (K^+^) is the most abundant cation in plants and an essential macronutrient [157,181]. Adequate K^+^ availability can increase tillering in plants, as evidenced by enhanced tillering in rice overexpressing *Oryza sativa High-Affinity* K^+^ *Transporter 5* (*OsHAK5*). In contrast, knockout of *OsHAK5* reduces tillers in rice [157], producing a phenotype resembling loss-of-function mutants of the auxin transporter *OsABCB14* [158], implying a potential interaction between K^+^ and auxin. Furthermore, driving expression of the *WUSCHEL-related homeobox* transcription factor gene *WOX11* using the promoter of *OsHAK16*, which encodes a low K^+^-induced K^+^ transporter, leads to increased effective tiller numbers in rice [159]. Together, these findings indicate that K^+^ availability modulates tillering at least through effects on K^+^ transporters like *OsHAK5* and associated transcriptional networks [159]. However, the specific molecular mechanisms by which K^+^ influences tiller development remain unclear. Further research is needed to elucidate the signaling pathways and gene regulatory networks through which K^+^ is perceived and transduced in axillary buds, promoting bud outgrowth.

#### 2.2.3. Water Availability Influences Bud Outgrowth

Tillering/branching processes are susceptible to drought stress, one of the most limiting factors affecting agricultural yields. Optimal water availability is critical for normal plant growth and development, including tillering. Tolerance to this stress is multigenic and complex in nature. Drought stress triggers specific alterations of gene-expression patterns in plant tissues [182]. For instance, the drought-inducible microRNA *miR393* was shown to be upregulated in Arabidopsis [183], and *miR393* also regulates tiller number increases in rice by modulating auxin signaling through auxin receptor genes like *OsAUX1* and *OsTIR1*. In addition, overexpression of *OsmiR393* downregulates the rice tillering inhibitor *OsTB1*, leading to increased tiller numbers [42] (Figure 3). Overexpression of *LRK2,* which encodes a leucine-rich receptor-like kinase gene, increases drought tolerance and tiller numbers in rice [160]. *Grain number*, *plant height*, and *heading date7* (*GHD7*) encoding a *CCT* (*CONSTANS, CONSTANS-LIKE, and TIMING OF CHLOROPHYLLA/B BINDING1*) domain protein regulates the rice flowering pathway and also contributes to rice yield potential. Overexpression of *GHD7* increases drought sensitivity, while knock-down of *GHD7* raises drought tolerance. Moreover, *GHD7* also regulates the plasticity of tillering by mediating the *PHYTOCHROME B-TEOSINTE BRANCHED1* pathway [161]. In summary, water availability or drought stress regulates tillering/branching in plants through effects on gene expression, microRNA levels, and modulation of hormonal signaling pathways like auxin signaling.

#### 2.2.4. Effects of Temperature on Tillering

Temperature is a critical factor influencing tillering in crops [184]. High temperatures caused by extreme weather events can reduce tiller numbers, as evidenced in *B*. *distachyon*. Tiller numbers declined linearly in B. *distachyon* from 24 to 36 °C at a rate of approximately one tiller for every 1.7 °C increase in temperature [185]. Genes involved in heat stress play an important role in tillering. For instance, in rice, *miR159* is downregulated by heat stress, and its overexpression increases heat sensitivity and significantly reduces tillering [186]. At the other extreme, chilling also detrimentally impacts tillering. Chilling tolerance is a complex agronomic trait governed by intricate genetic networks and signal transduction cascades. Mechanistic insights into cold-stress effects on tillering are emerging. For example, overexpression of *OsMADS57* maintains rice tiller growth under chilling stress. OsMADS57 directly binds and activates the defense gene *OsWRKY94* for cold-stress responses while suppressing its activity under normal temperatures [187]. Additionally, OsWRKY94 is directly targeted and repressed by the tillering inhibitor OsTB1 during chilling. *D14* transcription was directly promoted by OsMADS57 for suppressing tillering under chilling treatment, whereas *D14* was repressed for enhancing tillering under normal conditions [187] (Figure 3). Likewise, overexpression of *OsCYP19-4* results in cold-resistance phenotypes with significantly increased tiller numbers [188]. Ran is a small GTPase that involves various developments like nuclear assembly and cell-cycle control [189]. The levels of mRNA encoding OsRAN1 were greatly increased by chilling, and *OsRAN1* overexpression in *Arabidopsis* increased tiller numbers [162]. Elevated expression of *miR393* also improves chilling tolerance and tillering [190]. In summary, modulation of chilling-tolerance genes may benefit crop-breeding efforts to sustain tiller development under temperature extremes caused by climate change.

#### 2.2.5. Biotic Stresses Impact Tiller Bud Outgrowth

Biotic stress affects plant development, including tillering, fundamentally disrupting and depriving plants of the nutrients they rely on for survival. More specifically, biotic stresses caused by plant pathogens, insect pests, and parasitic organisms can impair growth and developmental processes such as tillering and branching (Figure 3). These biotic agents injure plant tissues both directly through feeding/infection and indirectly by inhibiting the uptake and utilization of water, nutrients, and photoassimilates required for plant growth. Pathogens, insects, and parasites disrupt key physiological processes like metabolism, resource allocation, and energy balance, ultimately reducing the plant’s capacity for producing new tillers or branches. Here, we take some examples to elucidate these processes. For instance, *Striga* is an obligate parasitic plant that can attach to host roots to deplete them of nutrients [191]. The rice cultivar Azucena, belonging to the japonica subspecies, exudes high SL levels and induces high germination of the root-parasitic plant *Striga hermonthica*. In contrast, Bala, an indica cultivar, is a low-SL producer, stimulates less *Striga* germination, and is highly tillered [192]. Plants relocate resources while fighting against pathogens and exhibit reduced tillering/branching. For example, the *UNI* gene encodes a coiled-coil nucleotide-binding leucine-rich repeat protein that belongs to the disease-resistance (R) protein family involved in pathogen recognition. The *uni-1D* mutation induces the upregulation of the pathogenesis-related gene while evoking some morphological defects like increased branches [22]. Further research into mitigation strategies against prevalent biotic agents would benefit efforts to secure plant growth and crop yields.

## 3. Concluding Remarks

Shoot branching is a highly intricate regulatory developmental program that involves the complex interplay of multiple genes, plant hormones, and environmental cues. A thorough understanding of the underlying mechanisms governing shoot branching/tillering is crucial for crop breeding and improving productivity (Video S1 and Figure 1). In this review, we provide a comprehensive overview of current advances in understanding the intricate regulatory mechanisms of shoot branching. We have highlighted key genes such as *TB1*, several phytohormones such as auxin, and environmental and internal inputs such as N, Pi, K^+^, light, water availability, and biotic stresses, underscoring the interplay of these components.

However, the complex regulation of shoot branching/tillering requires further investigation. The interplay of the various underlying internal and external cues is still not entirely understood. For example, further research is needed to fully understand how sugars collaborate with other factors to regulate shoot branching/tillering. Notably, plants with strong resistance to some pathogens often display fewer branches/tillers, but the exact mechanisms at play require further study. Importantly, AMs, which give rise to shoot branches/tillers, can also influence the patterns of panicles, hence affecting crop yields, an important factor in crop production and management [3,4,5]. With new achievements in understanding shoot branching, a comprehensive landscape of factors controlling shoot branching will be established. A better understanding of shoot branching will ultimately facilitate the control of the process.

## Figures and Tables

**Table 1:** List of genes related to lateral bud outgrowth. The table below lists a number of genes that have been identified and characterized for functions in the mentioned plant species.

Gene Names	Accession Numbers	Reported Species (Homolog)	Functional Annotation	References
*OsTB1*	*Os03g0706500*	Arabidopsis, rice, maize, pea, tomato	Transcription factor TCP family	[6,29,30,31]
*OsSPL14* (*IPA1*), *OsSPL15*	*Os08g0509600*, *Os08g0513700*	Rice, Arabidopsis	SQUAMOSA promoter binding protein-like transcription factors	[5,32,33]
*AXR1*	*AT1G05180*	Arabidopsis	a subunit of the RUB1 activating enzyme	[34]
*YUCCA*	*AT4G32540*	Arabidopsis	A flavin monooxygenase-like enzyme, auxin biosynthesis	[35]
*PIN1*	*Os02g0743400*	Rice	an auxin transporter	[36]
*OsPIN5b*	*Os09g0505400*	Rice	an auxin transporter	[37]
*MKK7*	*AT1G18350*	Arabidopsis	MAP kinase kinase7	[38]
*MKK6*	*AT5G56580*	Arabidopsis	MAP kinase kinase 6	[39]
*TIR1*	*Os05g0150500*, *AT1G72930*	Rice, Arabidopsis	auxin receptor	[9,40]
*IAA12*	*AT1G04550*	Arabidopsis	an auxin-responsive gene	[41]
*AFB2*	*Os04g0395600*	Rice	Auxin signaling f-box 2	[42]
*RUB1*	*AT1G31340*	Arabidopsis	A ubiquitin-related protein	[9]
*D27*	*Os11g0587000*	Rice	An iron-containing protein	[12,43]
*CCD7*/*MAX3*	*AT2G44990*, *Os04g0550600*	Arabidopsis, rice	carotenoid cleavage dioxygenases	[44]
*CCD8*/*MAX4*	*AT4G32810*, *Os01g0746400*	Arabidopsis, rice	carotenoid cleavage dioxygenases	[45]
*MAX1*	*AT2G26170*	Arabidopsis	Belonging to the CYP711A cytochrome P450 family	[46]
*MAX2*/*D3*	*AT2G42620*, *Os06g0154200*	Arabidopsis, rice	Belonging to a member of the F-box leucine-rich repeat family	[46]
*D14*	*Os03g0203200*, *AT3G03990*	Rice, Arabidopsis	An alpha/beta hydrolase	[47]
*D53*	*Os11g0104300*	Rice	The substrate of SCF-D3 ubiquitin complex	[48]
*SMXL6*, *SMXL7*, *SMXL8*	*AT1G07200*, *AT2G29970*, *AT2G40130*	Arabidopsis	D53-like proteins	[48,49]
*IPT*	*AT3G23630*	Arabidopsis	An isopentenyl transferase	[50]
*SPS*	*AT1G16410*	Arabidopsis	Belonging to a member of CYP79F	[51]
*AMP1*	*AT3G54720*	Arabidopsis	A glutamate carboxypeptidase	[8]
*PsCKX2*	*LOC127082854*	Pea	Cytokinin dehydrogenase 6-like	[52]
*NCED3*	*AT3G14440*	Arabidopsis	A 9-cis-epoxycarotenoid dioxygenase	[53]
*ABA2*	*AT1G52340*	Arabidopsis	A cytosolic short-chain dehydrogenase	[54]
*HB21*, *HB40*, *HB53*	*AT2G02540*, *AT4G36740*, *AT5G66700*	Arabidopsis	Homeobox proteins	[14]
*SLR1*	*Os03g0707600*	Rice	A DELLA protein	[32]
*BES1*	*AT1G19350*	Arabidopsis	A transcription factor	[55]
*MOC2*	*Os01g0866400*	Rice	A cytosolic fructose 1,6-bisphosphatase	[56]
*OsNPF7.7*	*Os10g0579600*	Rice	One nitrate transporter	[57]
*TaNAC2-5A*	*AY625683*	Wheat	A transcription factor	[58]
*OsMADS57*	*Os02g0731200*	Rice	A MADS transcription factor 57	[59]
*OsBZR1*, *BES1*	*Os07g0580500*, *AT1G19350*	Rice, Arabidopsis	A key transcription factor involved in brassinosteroid (BS) signaling	[60]
*DLT*	*Os06g0127800*	Rice	A GRAS protein	[61]
*GSK2*	*Os05g0207500*	Rice	A conserved glycogen synthase kinase 3-like kinase	[61]
*RLA1*	*Os05g0389000*	Rice	An APETALA2 (AP2) DNA binding domain protein	[62]
*BRI1/D61*	*Os01g0718300*	Rice	A BR receptor	[61]

**Table 2:** List of genes related to lateral bud outgrowth response to environmental inputs. The table below lists a number of genes that have been identified and characterized for functions in the mentioned plant species.

Gene Names	Accession Numbers	Reported Species (Homolog)	Functional Annotation	References
*PHYB*	*LOC8081072*	*Sorghum bicolor*	Phytochrome B	[149]
*SbTB1*	*LOC8062930*	*Sorghum bicolor*	Belonging to transcription factor of the TCP family	[149]
*NCED3*	*AT3G14440*	Arabidopsis	A 9-*cis*-epoxycarotenoid dioxygenase	[53]
*ABA2*	*AT1G52340*	Arabidopsis	A cytosolic short-chain dehydrogenase/reductase	[53]
*OsNPF7.7*	*Os10g0579600*	Rice	Belonging to the peptide transporter (PTR) gene family	[57]
*OsNR2*	*Os02g0770800*	Rice	NADH/NADPH-dependent NO_3_^−^ reductase 2	[150]
*NGR5*	*Os05g0389000*	Rice	One APETALA2-domain transcription factor	[151]
*PRC2*	*Os03g0108700*	Rice	A polycomb repressive complex 2-associated coiled-coil protein	[151]
*D14*	*Os03g0203200*	Rice	A strigolactone receptor	[151]
*SPL14*	*Os08g0509600*	Rice	A squamosa promoter-binding-like transcription activator	[151]
*OsDEP1*	*Os09g0441900*	Rice	One unknown phosphatidylethanolamine-binding protein (PEBP)-like domain protein	[152]
*OsAFB2*	*Os04g0395600*	Rice	An auxin receptor	[153]
*OsTIR1*	*Os05g0150500*	Rice	A F-Box auxin receptor protein	[153]
*OsTCP19*	*Os06g0226700*	Rice	A class-I TCP transcription factor	[154]
*TaNAC2-5A*	*LOC606326*	Wheat	NAC domain-containing protein 2	[58]
*OsMADS57*	*Os02g0731200*	Rice	A MADS-box transcription factor	[59]
*OsPHR2*	*Os07g0438800*	Rice	A MYB-CC family protein	[155]
*NSP1*	*Os03g0408600*	Rice	A GRAS-domain transcription factor	[156]
*NSP2*	*Os03g0263300*	Rice	A GRAS-domain transcription factor	[156]
*OsHAK5*	*Os01g0930400*	Rice	A potassium transporter	[157]
*OsABCB14*	*Os04g0459000*	Rice	An auxin transport	[158]
*WOX11*	*Os07g0684900*	Rice	A WUSCHEL-related homeobox protein	[159]
*OsHAK16*	*Os03g0575200*	Rice	A high-affinity potassium transporter	[159]
*OsAUX1*	*Os01g0856500*	Rice	An auxin transporter	[42]
*LRK2*	*Os02g0154000*	Rice	A leucine-rich repeat receptor-like kinase	[160]
*GHD7*	*Os07g0261200*	Rice	A CCT(CONSTANS, CONSTANS-LIKE, and TIMING OF CHLOROPHYLL A/B BINDING1) domain protein	[161]
*OsRAN1*	*Os01g0611100*	Rice	A small GTPase	[162]

## Data Availability

No new data were created or analyzed in this study. Data sharing is not applicable to this article.

## Supplementary Materials

The following supporting information can be downloaded at: https://www.mdpi.com/article/10.3390/plants12203628/s1, Video S1: Illustration of the dynamic process of tiller formation and comparisons between plants with normal and defective tiller bud outgrowth.
